# Supplementary material for: The Small RNA Universe of Capitella teleta
Source: Front Mol Biosci. 2022 Feb 25;9:802814. doi: 10.3389/fmolb.2022.802814 (PMC8915122; doi:10.3389/fmolb.2022.802814)
Supplement: Supplementary file 1 [file DataSheet1.ZIP › Supplement/known/CAPTEscaffold_296_17126.pdf]

Provisional ID	:	CAPTEscaffold_296_17126
Score total	:	14371.4
Score for star read(s)	:	3.9
Score for read counts	:	14361.4
Score for mfe	:	1.5
Score for randfold	:	1.6
Score for cons. seed	:	3
Total read count	:	28181
Mature read count	:	28132
Loop read count	:	0
Star read count	:	49

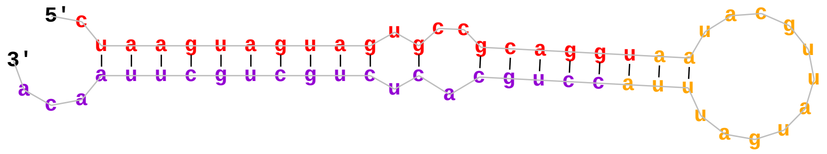

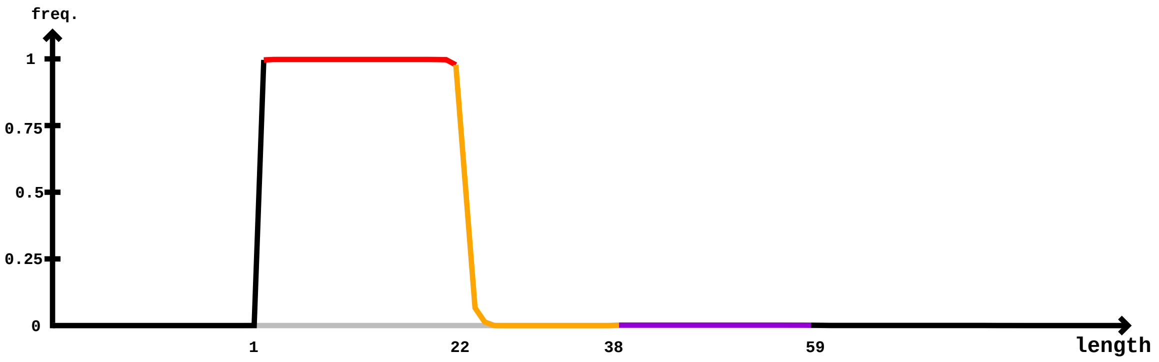


```
                        Mature                           Star
5'- ugcaucucacugugacagugcuaaguaguagugccgcagguaauacguuaugauuuaccugcacucugcugcuuaacacaagcacgcgugaauaacaacaucauaacgca  -3'   obs
    ugcaucucacugugacagugcuaaguaguagugccgcagguaauacguuaugauuuaccugcacucugcugcuuaacacaagcacgcgugaauaacaacaucauaacgca        exp
    ugcaucucacugugacagugcuaaguaguagugccgcagguaauacguuaugauuuaccugcacucugcugcuuaacacaagcacgcgugaauaacaacaucauaacgca        known
    ......((((((((...(((.((((((((((.(..((((((((..........)))))))).).)))))))))).)))...)))).))))....................   reads   mm   sample
    ...................Ccuaaguaguagugccgcaggua....................................................................   2       1    seq
    ....................cuaaguaguagugccgca........................................................................   9       0    seq
    ....................cuaaguaguagugccgcag.......................................................................   18      0    seq
    ....................cuaaguaguagugccgcagU......................................................................   3       1    seq
    ....................cuaaguaguagugcAgcagg......................................................................   1       1    seq
    ....................cAaaguaguagugccgcagg......................................................................   1       1    seq
    ....................cuaaguaguagugccgcaAg......................................................................   1       1    seq
    ....................cuaaguaguagugccgcagg......................................................................   530     0    seq
    ....................cuaaguaAuagugccgcagg......................................................................   1       1    seq
    ....................cCaaguaguagugccgcagg......................................................................   1       1    seq
    ....................Guaaguaguagugccgcagg......................................................................   1       1    seq
    ....................cuaaguaguagugccgcagA......................................................................   1       1    seq
    ....................Nuaaguaguagugccgcagg......................................................................   1       1    seq
    ....................cuaaguaguaUugccgcagg......................................................................   1       1    seq
    ....................cuaaguaguagugccgcaUg......................................................................   1       1    seq
    ....................cuaaguaCuagugccgcaggu.....................................................................   5       1    seq
    ....................cuaaguaguagugccAcaggu.....................................................................   2       1    seq
    ....................cuGaguaguagugccgcaggu.....................................................................   1       1    seq
    ....................cuaaguaguagugccgcaggC.....................................................................   2       1    seq
    ....................cuaaguaguagugccgcagUu.....................................................................   1       1    seq
    ....................cuaaguaguagugcUgcaggu.....................................................................   7       1    seq
    ....................cuaaguaguaguAccgcaggu.....................................................................   1       1    seq
    ....................cuaaguaguagugccgcUggu.....................................................................   2       1    seq
    ....................Auaaguaguagugccgcaggu.....................................................................   16      1    seq
    ....................cuaaguaguagugccgcaggu.....................................................................   11506   0    seq
    ....................cuaaguUguagugccgcaggu.....................................................................   1       1    seq
    ....................cuaaguagAagugccgcaggu.....................................................................   1       1    seq
    ....................cuaaguaguagGgccgcaggu.....................................................................   1       1    seq
    ....................cuaaguaguagugccUcaggu.....................................................................   2       1    seq
    ....................cuaaguaguagugAcgcaggu.....................................................................   2       1    seq
    ....................cNaaguaguagugccgcaggu.....................................................................   1       1    seq
    ....................Nuaaguaguagugccgcaggu.....................................................................   4       1    seq
```

```
                    Mature                                Star
ugcaucucacugugacagugcuaaguaguagugccgcagguaauacguuaugauuuaccugcacucugcugcuuaacacaagcacgcgugaauaacaacaucauaacgca
....................cuaaCuaguagugccgcaggu.....................................................................	1	1	seq
....................cuaagAaguagugccgcaggu.....................................................................	6	1	seq
....................Guaaguaguagugccgcaggu.....................................................................	1	1	seq
....................cuaaguaUuagugccgcaggu.....................................................................	1	1	seq
....................cuaaguaguagugccgcaggA.....................................................................	112	1	seq
....................cuaaguaguagugccgcaggG.....................................................................	10	1	seq
....................cuaaguagGagugccgcaggu.....................................................................	2	1	seq
....................cCaaguaguagugccgcaggu.....................................................................	1	1	seq
....................cuaaguaguagAgccgcaggu.....................................................................	7	1	seq
....................cuaaguaguagugcAgcaggu.....................................................................	2	1	seq
....................cuaaguGguagugccgcaggu.....................................................................	1	1	seq
....................cAaaguaguagugccgcaggu.....................................................................	20	1	seq
....................cuaaguaguagugccgAaggu.....................................................................	4	1	seq
....................cuaaguaguagugccCcaggu.....................................................................	1	1	seq
....................cuaaAuaguagugccgcaggu.....................................................................	5	1	seq
....................cuaaguaguaUugccgcaggu.....................................................................	1	1	seq
....................cuaaguaguagugccgcGggu.....................................................................	4	1	seq
....................cuaaguaguagugccgUaggu.....................................................................	2	1	seq
....................cuaaguaguagugccgcaAgu.....................................................................	5	1	seq
....................cuaaguaguagugccgGaggu.....................................................................	1	1	seq
....................Uuaaguaguagugccgcaggu.....................................................................	8	1	seq
....................cuaaguagCagugccgcaggua....................................................................	1	1	seq
....................Nuaaguaguagugccgcaggua....................................................................	2	1	seq
....................cuaaguUguagugccgcaggua....................................................................	3	1	seq
....................cuaaguaguaUugccgcaggua....................................................................	1	1	seq
....................cuaaguaguagCgccgcaggua....................................................................	5	1	seq
....................cCaaguaguagugccgcaggua....................................................................	1	1	seq
....................cuaaguaguGgugccgcaggua....................................................................	5	1	seq
....................cuaaguagGagugccgcaggua....................................................................	1	1	seq
....................cuaUguaguagugccgcaggua....................................................................	1	1	seq
....................cuaaguaguagugccgcagUua....................................................................	2	1	seq
....................cuaaguaguagugccgcaUgua....................................................................	2	1	seq
....................cAaaguaguagugccgcaggua....................................................................	14	1	seq
....................Uuaaguaguagugccgcaggua....................................................................	8	1	seq
....................cuaaguaguagugccgGaggua....................................................................	1	1	seq
....................cuaaguaguaCugccgcaggua....................................................................	1	1	seq
....................cuaaguaguagugccgcUggua....................................................................	1	1	seq
....................cuaagAaguagugccgcaggua....................................................................	6	1	seq
....................cGaaguaguagugccgcaggua....................................................................	2	1	seq
....................cuCaguaguagugccgcaggua....................................................................	1	1	seq
....................cuaaguaguagugccAcaggua....................................................................	2	1	seq
....................cuaGguaguagugccgcaggua....................................................................	1	1	seq
....................cuaaguaguagugccgcagguU....................................................................	2465	1	seq
....................cuaaguaguagugccgcaggAa....................................................................	3	1	seq
....................cuaaguaguagugcAgcaggua....................................................................	6	1	seq
....................cuaaguaguagugcUgcaggua....................................................................	3	1	seq
....................cuGaguaguagugccgcaggua....................................................................	4	1	seq
....................Auaaguaguagugccgcaggua....................................................................	6	1	seq
....................cuaaguagAagugccgcaggua....................................................................	5	1	seq
....................cuaaguaguagugccgcagguC....................................................................	958	1	seq
....................cuaagCaguagugccgcaggua....................................................................	1	1	seq
....................cuaaguaguagugccgcaggua....................................................................	10342	0	seq
....................cuaaguaguagugccgcGggua....................................................................	2	1	seq
....................cuaaguaCuagugccgcaggua....................................................................	2	1	seq
....................cuaagGaguagugccgcaggua....................................................................	1	1	seq
....................cuaaguaguagugAcgcaggua....................................................................	3	1	seq
....................cuaaguaguagugccgcaAgua....................................................................	1	1	seq
....................cuaaUuaguagugccgcaggua....................................................................	1	1	seq
....................cuaaguaguagugccgUaggua....................................................................	1	1	seq
....................cuaaguaguagugccgcagguG....................................................................	5	1	seq
....................cuaaguaguaguAccgcaggua....................................................................	1	1	seq
....................cuaaguaguagAgccgcaggua....................................................................	5	1	seq
....................cuaaguaguagugccgcaggCa....................................................................	10	1	seq
....................cuaaguaguUgugccgcaggua....................................................................	1	1	seq
....................cuaaguGguagugccgcaggua....................................................................	2	1	seq
....................cuaaguaguagugccgcagguUa...................................................................	7	1	seq
....................cuaaguaguaCugccgcagguaa...................................................................	1	1	seq
....................cuaaguaguagugccgcagguaU...................................................................	971	1	seq
....................cuaaguaguagugccAcagguaa...................................................................	1	1	seq
....................cuaaguagAagugccgcagguaa...................................................................	1	1	seq
```

```
                    Mature                                Star
ugcaucucacugugacagugcuaaguaguagugccgcagguaauacguuaugauuuaccugcacucugcugcuuaacacaagcacgcgugaauaacaacaucauaacgca
....................cuaaguaguagugccgcagguaa...................................................................  474  0  seq
....................cuaaguaguagugccgcagguaG...................................................................  6    1  seq
....................cuaaguaguagugccgcagguaC...................................................................  23   1  seq
....................cuaaguaguagugccgcagguaau..................................................................  76   0  seq
....................cuaaguaguagugccgcagguaaA..................................................................  43   1  seq
....................cuaaguaguagugccgcagguUau..................................................................  2    1  seq
....................cuaaguaguagugccgcagguaUu..................................................................  259  1  seq
....................cuaaguaguagugccgcagguaCu..................................................................  1    1  seq
....................cuaaguaguagugccgcagguaaAa.................................................................  2    1  seq
....................cuaaguaguagugccgcagguaauU.................................................................  1    1  seq
....................cuaaguaguagugccgcagguaaGa.................................................................  3    1  seq
.....................uaaguaguagugccgcaggu.....................................................................  15   0  seq
.....................uaaguaguagugccgcaggua....................................................................  21   0  seq
.....................uaaguaguagugccgcagguU....................................................................  8    1  seq
.....................uaaguaguagugccgcagguaa...................................................................  2    0  seq
.....................uaaguaguagugccgcagguaU...................................................................  3    1  seq
......................aaguaguagugccgcaggua....................................................................  1    0  seq
........................................................accugcacucugcugcuua...................................  1    0  seq
........................................................accugcacucugcugcuuaaca................................  2    0  seq
........................................................accugcacucugcugcuuaGca................................  1    1  seq
.........................................................ccugcacucugcugcuua...................................  2    0  seq
.........................................................ccugcacucugcugcuuaac.................................  9    0  seq
.........................................................ccugcacucugcugcuuaacC................................  9    1  seq
.........................................................ccugcacucugcugcuuaaca................................  22   0  seq
.........................................................ccugcacucugcugcuuaacU................................  3    1  seq
..............................................................................cUagcacgcgugaauaac..............  6    1  seq
..............................................................................cUagcacgcgugaauaaca.............  2    1  seq
..............................................................................cUagcacgcgugaauaacaac...........  2    1  seq
..............................................................................cUagcacgcgugaauaacaacau.........  1    1  seq
```
